# Enhancing Stability of *Boesenbergia rotunda* Bioactive Compounds: Microencapsulation via Spray-Drying and Its Physicochemical Evaluation

**DOI:** 10.3390/foods14152699

**Published:** 2025-07-31

**Authors:** Fahmi Ilman Fahrudin, Suphat Phongthai, Pilairuk Intipunya

**Affiliations:** 1 Doctoral Degree Program in Food Science and Technology, Chiang Mai University, Chiang Mai 50100, Thailand; fahmi_fahrudin@cmu.ac.th; 2 Division of Food Science and Technology, Faculty of Agro-Industry, Chiang Mai University, Chiang Mai 50100, Thailand; suphat.phongthai@cmu.ac.th; 3 Cluster of High Value Products from Thai Rice and Plants for Health, Faculty of Agro-Industry, Chiang Mai University, Chiang Mai 50100, Thailand

**Keywords:** fingerroot, microencapsulation, encapsulation efficiency, stability, bioactive contents, powder properties

## Abstract

This study aimed to microencapsulate *Boesenbergia rotunda* (fingerroot) extract using maltodextrin (MD) and gum arabic (GA) as wall materials via spray-drying to improve powder physicochemical properties and protect bioactive compounds. MD and GA were employed as wall materials in varying ratios (MD:GA of 1:0, 0:1, 1:1, 2:1, 1:2) to evaluate their effects on the physicochemical properties of the resulting microcapsules. Spray-dried microcapsules were evaluated for morphology, flowability, particle size distribution, moisture content, hygroscopicity, solubility, encapsulation efficiency, major bioactive compound retention, and thermal stability. The extract encapsulation using MD:GA at 1:1 ratio (MD1GA1) demonstrated a favorable balance, with high solubility (98.70%), low moisture content (8.69%), low hygroscopicity (5.08%), and uniform particle morphology, despite its moderate EE (75.06%). SEM images revealed spherical particles with fewer surface indentations in MD-rich formulations. Microencapsulation effectively retained pinostrobin and pinocembrin in all formulations with pinostrobin consistently retained at a higher value, indicating its higher stability. The balanced profile of physical and functional properties of fingerroot extract with MD1GA1 microcapsule makes it a promising candidate for food and nutraceutical applications.

## 1. Introduction

Thailand is home to a rich diversity of medicinal rhizomatous plants, many of which are integral to both traditional medicine and local cuisine. Species from the *Zingiberaceae* family are widely recognized for their pharmacological potential and are commonly used in herbal remedies for treating inflammation, infections, and gastrointestinal disorders. Among the various medicinal rhizomes native to Thailand, *Boesenbergia rotunda* (L.) Mansf., commonly referred to as fingerroot, has gained increasing scientific interest due to its wide spectrum of bioactivities and longstanding role in traditional health practices. As research continues to explore its pharmacological properties, fingerroot has emerged as a promising botanical resource for developing novel health-promoting products in the food and pharmaceutical industries. Traditionally, fingerroot has been employed to treat various ailments, including digestive disorders, respiratory infections, and inflammatory conditions, underscoring its value in ethnomedicine [1]. In addition to its therapeutic uses, it is widely appreciated as a culinary ingredient that enhances the flavor and nutritional quality of foods. The therapeutic potential of fingerroot is primarily attributed to its rich profile of bioactive phytochemicals, particularly flavonoids such as pinostrobin and pinocembrin. These compounds have been reported to possess various biological activities, including antioxidant, anti-inflammatory, antimicrobial, and anticancer effects [2]. Their ability to mitigate oxidative stress and modulate key biological pathways makes them attractive candidates for use in functional foods, nutraceuticals, and pharmaceutical formulations [3]. However, the direct application of fingerroot extracts has faced significant limitations due to the chemical instability of these bioactive constituents. When exposed to environmental factors such as heat, light, oxygen, and moisture, they are susceptible to degradation, which diminishes their functional efficacy [4]. Moreover, their poor water solubility and low bioavailability hinder their absorption and therapeutic performance in vivo [5]. These challenges highlight the need for effective protective strategies to stabilize and deliver fingerroot bioactives in a bioavailable form.

Microencapsulation is a promising approach to address these issues by entrapping sensitive bioactive compounds within a protective matrix. This technique enhances physicochemical stability, solubility, and controlled release of encapsulated ingredients, improving their shelf life and functional performance [5]. Among the various encapsulation techniques, spray-drying is particularly attractive due to its cost-efficiency, scalability, rapid processing time, and ability to produce powders with tailored characteristics [6].

The success of spray-drying depends significantly on selecting appropriate wall materials. Maltodextrin (MD) and gum arabic (GA) are commonly used carriers due to their film-forming ability, emulsification properties, low viscosity, and compatibility with bioactive compounds. These wall materials protect sensitive ingredients from environmental degradation during drying and storage, while also influencing powder morphology, solubility, and encapsulation efficiency [7]. Optimizing the combination and ratio of these wall materials can significantly improve the functional properties of the encapsulated powder. While several studies have investigated the encapsulation of various plant extracts using spray-drying, comprehensive data on the microencapsulation of *Boesenbergia rotunda*, especially concerning the physicochemical and thermal behavior of different MD and GA formulations, remain limited. Therefore, this study aimed to optimize the microencapsulation of fingerroot extract using spray-drying by evaluating the effects of different MD:GA ratios on powder characteristics, encapsulation efficiency, bioactive retention, and thermal stability.

This research provides novel insights into how wall material composition influences the protective capacity and functionality of encapsulated fingerroot bioactives. The findings contribute valuable knowledge for developing stable, functional powder ingredients suitable for applications in food, beverage, and nutraceutical industries.

## 2. Materials and Methods

### 2.1. Core Materials Preparations

The Thai fingerroot extract was obtained using optimized supercritical carbon dioxide (CO_2_) fluid extraction (SFE) conditions, performed on a Speed™ SFE-2 system (Applied Separations, Allentown, PA, USA). The extraction was conducted using 20 g of freeze-dried fingerroot powder, which had been previously purchased, stored at 4 °C for 24 h, cleaned, sliced, freeze-dried at −40 °C under 20 Pa for 24 h, grinded and sieved through a 45-mesh screen to ensure uniform particle size. The extraction was performed at 250 bar and 45 °C, with a CO_2_ flow rate of 2 L/min and 100% *v*/*w* food-grade ethanol as a co-solvent, following the conditions established by Fahrudin et al. [8]. The collected extract was concentrated to dryness using a rotary vacuum evaporator and then reconstituted in 95% ethanol to a final volume of 20 mL. This standardized liquid extract served as the core material for emulsification and microencapsulation.

The concentrated extract was subsequently blended with the wall material solution at a 1:1 (*w*/*w*) core-to-wall ratio, ensuring consistent loading across all formulations. The wall material solutions were prepared at 20% *w*/*v* total solids with varying MD:GA ratios (1:0, 0:1, 1:1, 2:1, and 1:2). Based on this ratio, approximately 0.5 g of fingerroot extract was used per 1 g of wall material, depending on the total solid concentration. These proportions were selected to achieve a stable emulsion and efficient encapsulation while maintaining consistent total solid content across samples.

### 2.2. Preparation of the Wall Materials

The maltodextrin used in this study had a dextrose equivalent (DE) of 10, and was food-grade with no additives (Yok Co., Ltd., Chiang Mai, Thailand). The gum arabic was obtained from *Acacia senegal* and was food-grade, and contained approximately 2.5% protein, with a 10% solution viscosity of ~20 mPa·s (Union Science Trading Co., Ltd., Chiang Mai, Thailand). The feed formulations for spray-drying were prepared by dissolving wall materials, maltodextrin (MD) and gum arabic (GA) in 300 mL of distilled water. A total of 80 g of carrier agent was used in each formulation, corresponding to a fixed concentration of 20% (*w*/*v*) based on the total solution weight. The MD-to-GA ratios were varied as follows: 1:0, 0:1, 1:1, 2:1, and 1:2, as detailed in Table 1. The wall materials were stirred until fully dissolved to form a homogeneous solution. After achieving complete dissolution of the carrier agents, core material was added to each solution, resulting in a total sample weight of approximately 400 g. The total soluble solids (TSS) were fixed at 20 °Brix for all formulations to ensure comparability. The selected ratios were adapted from a previous study on fingerroot encapsulation, which employed total wall material concentrations ranging from 20% to 30% *w*/*v* and component ratios of 1:0, 0:1, 1:1, 2:1, and 1:2 [9,10].

The mixture was then homogenized at 8000 rpm for 10 min using a high-speed homogenizer (Homogenizer, T25 Digital Ultra Turrax^®^, IKA Works Co., Ltd., Bangkok, Thailand) to ensure uniform emulsification of the extract within the carrier matrix. Following homogenization, the emulsions were continuously stirred using a magnetic stirrer to maintain dispersion and prevent separation. Finally, the emulsions were rapidly cooled to below 10 °C within 10 min to stabilize the system before spray-drying.

### 2.3. Microencapsulation by Spray-Drying

The extract was encapsulated using a spray-dryer under optimized conditions based on previous studies, with some adjustments [9]. A mini spray-dryer (Büchi B-290, Büchi Labortechnik AG, Flawil, Switzerland) equipped with a 1.4 mm nozzle was used to produce the microcapsule powder. The inlet air temperature was set at 150 °C, and the outlet temperature was maintained at 90 °C. The feed solution was kept at 28 °C, with a flow rate of 3 mL/min, and the aspirator was set to 80%. Prior to and after each run, the system was flushed with distilled water for 10 min to ensure cleanliness and calibration. The resulting microcapsule powders were collected, weighed, and stored in airtight aluminum bags for further analysis.

### 2.4. Physical Property Analysis

#### 2.4.1. Powder Flow-Related Properties

The loose bulk density and tapped bulk density of the spray-dried powders were determined to assess flowability using the method of Beristain et al. [11]. Approximately 2.0 g of powder was gently transferred into a 10 mL graduated cylinder without tapping, and the volume was recorded. The loose bulk density (g/mL) was calculated as the ratio of sample weight to the untapped volume, as shown in Equation (1).

(1) $$\mathrm{Loose}\;\mathrm{bulk}\;\mathrm{density}\;(g/\mathrm{mL})=\frac{Mass\;of\;the\;powder\;(g)}{Bulk\;volume\;(mL)}$$

For tapped bulk density, the same cylinder was tapped manually and consistently 100 times from a fixed height until no further volume change occurred. The tapped volume was recorded, and the tapped bulk density (g/mL) was calculated accordingly as shown in Equation (2).

(2) $$\mathrm{Tapped}\;\mathrm{bulk}\;\mathrm{density}\;(g/\mathrm{mL})=\frac{Mass\;of\;the\;powder\;(g)}{Tapped\;volume\;(mL)}$$

The loose bulk density and tapped bulk density measurements were used to determine the Hausner ratio and Carr index utilizing Equations (3) and (4), respectively. Table 2 shows the relationship between the Carr index and the Hausner ratio with powder flowability.

(3) $$\mathrm{Hausner}\;\mathrm{ratio}=\frac{Tapped\;bulk\;density\;(g/mL)}{Loose\;bulk\;density\;(g/mL)}$$

(4) $$\mathrm{Carr}\;\mathrm{index}=\frac{Tapped\;bulk\;density\left(g/mL\right)-Loose\;bulk\;density\;(g/mL)}{Tapped\;bulk\;density\;(g/mL)}$$

#### 2.4.2. Hygroscopicity

This method evaluated the water absorption capacity (hygroscopicity) of the microparticles, following a modified protocol by Arebo et al. [12]. Precisely two grams of the sample powder was evenly spread onto clean 9 cm Pyrex glass Petri dishes. These dishes were placed in a desiccator maintained at 25 °C and 74% relative humidity, using a saturated NaCl solution, and left for 120 min. Afterward, the samples were removed and immediately weighed. The increase in weight was recorded and used to calculate hygroscopicity according to Equation (5).

(5) $$\mathrm{Hygroscopicity}\;(\%)=\frac{\left(Final\;weight-Initial\;weight\right)}{\left(Initial\;weigth\right)}\times 100$$

#### 2.4.3. Degree of Powder Caking

Following the determination of hygroscopicity, the degree of caking powder is determined using a method of Bashir et al. [13] with some adjustments. The wet sample was placed in a vacuum oven at 70 °C for approximately 16 h. After cooling, the dried sample was weighed and transferred to a 40-mesh (0.4 mm) sieve. The sieve was subjected to shaking for 5 min using a shaking apparatus. The weight of the powder retained in the sieve was then measured. The degree of caking was calculated as follows, using Equation (6).

(6) $$\mathrm{Powder}\;\mathrm{caking}\;(\%)=\frac{\left(100\times A\right)}{B}$$

where A is the amount of powder retained on the sieve, and B is the initial amount of powder.

#### 2.4.4. Microcapsule Solubility

The solubility of the microcapsules was assessed using Balci-Torun et al.’s [14] methods. A 10 mg sample was introduced into 50 mL of distilled water and stirred for 5 min using a magnetic stirrer. Subsequently, the mixture underwent centrifugation at 5000 rpm for 5 min. The supernatant was carefully transferred to Petri dishes and maintained at 70 °C for approximately 16 h. The percentage solubility was then calculated based on the weight difference.

#### 2.4.5. Particle Size Distribution

The analysis focused on evaluating the particle size, polydispersity index (PDI) and D[4,3] of the spray-dried powder to determine the uniformity of the microparticles. The method was adapted from Caballero-Román et al. [15] with slight modifications. Specifically, 500 mg of spray-dried powder was dispersed in 10 mL of isopropanol (refractive index 1.390, AR grade, RCI Labscan, Bangkok, Thailand). From this dispersion, 3 mL of the microparticle suspension (at 5% *w*/*v*) was transferred into a square glass cuvette for measurement using the Malvern Zetasizer ZSU5700 (Malvern Panalytical Ltd., Worcestershire, UK). The instrument was calibrated and operated at 25 °C, and each sample was measured in triplicate to ensure accuracy and consistency.

The volume-weighted mean diameter (D[4,3]), also known as the De Brouckere mean diameter, represents the average particle size weighted by volume and is calculated by dividing the sum of the fourth power of particle diameters by the sum of their third power. This parameter is especially sensitive to the presence of larger particles due to the cubic volume relationship and is widely used in laser diffraction-based measurements where volume or mass-based behavior is critical. D[4,3] provides insight into powder behavior during processing, particularly regarding solubility, sedimentation, and flow.

The polydispersity index (PDI) was also recorded as a dimensionless measure of the width of the particle size distribution. Lower PDI values (typically <0.2) indicate a more monodisperse system, while higher values reflect greater heterogeneity, which can influence the physical stability and reproducibility of spray-dried powders.

#### 2.4.6. Powder Morphology Analysis

The morphological characteristics of powders produced under optimal conditions were analyzed using scanning electron microscopy (SEM). Before examination, the samples were coated with a 10 nm layer of gold and observed using SEM (model JEOL JSM-6610LV, JEOL Ltd., Tokyo, Japan). The machine was configured with a secondary electron detector (SED) set at 15.0 kV, a working distance (WD) of 10.3 mm, and a high vacuum mode. Imaging was performed at three zoom levels, ×3 k, ×6 k, and ×9 k, to achieve varying resolutions.

### 2.5. Physicochemical Property Analysis

#### 2.5.1. Determination of Water Activity

The powder’s water activity was assessed using a Decagon AquaLab Pre water activity analyzer (Decagon Devices, Inc., Pullman, WA, USA). Approximately 5 mg of powder was placed in the sample holder, and all measurements were conducted in duplicate on the samples immediately after the drying process at 25 °C.

#### 2.5.2. Moisture Content of Powder

The moisture can was dried in a hot air oven at 105 °C for 3 h. After cooling in a desiccator, the can’s weight was recorded. Approximately 1–2 g of the sample was then placed into the dried moisture can, which was subsequently returned to the hot air oven at 105 °C for 6 h or until the weight remained constant [16]. The moisture content was calculated using Equation (7).

(7) $$\mathrm{Moisture}\;\mathrm{Content}\;(\%\;\mathrm{db})=\frac{\left(A-B\right)}{\left(B\right)}\times 100$$

where A represents the weight of the sample before drying (g), and B represents the weight after drying (g).

#### 2.5.3. Total Phenolic and Flavonoid Contents

To evaluate the total phenolic content (TPC) of the samples, a colorimetric assay based on the Folin–Ciocalteu reagent was employed. The determination of TPC was performed using a modified Folin–Ciocalteu colorimetric method as outlined by Velioglu et al. [17]. In this procedure, 500 μL of the extract was mixed with 2.5 mL of 10% (*v*/*v*) Folin–Ciocalteu reagent and allowed to stand for 5 min at room temperature. Following this, 2 mL of 7.5% (*v*/*v*) sodium carbonate solution was added, and the mixture was incubated in the dark for 45 min at 28 °C to allow full color development. Absorbance was measured at 760 nm using a GENESYS™ 180 UV–Visible spectrophotometer (Thermo Fisher Scientific, Waltham, MA, USA). A calibration curve was prepared using standard gallic acid solutions, and results were expressed as milligrams of gallic acid equivalents (mg GAE) per gram of microcapsule (powder). The equation is mentioned below:

(8) $$\mathrm{TPC}\;(\mathrm{mg}\;\mathrm{GAE}/g\;\mathrm{powder})\;=\;\frac{\left(GA\times EV\right)}{\left(ME\right)}$$

where GA is gallic acid (mg/mL) as a standard, EV is the extract volume (mL), and ME is mass of the extract microcapsule powder.

Quantification of total flavonoid content (TFC) was carried out using an aluminum chloride-based colorimetric method. The analysis adapted the method described by Gatti et al. [18], with quercetin serving as the reference compound. A calibration curve was established using quercetin standards dissolved in ethanol at varying concentrations. For each reaction, 100 μL of the quercetin standard (or sample extract) was mixed with 500 μL of distilled water, followed by the addition of 100 μL of 5% (*w*/*v*) sodium nitrate. After a 6-min incubation period at room temperature, 150 μL of 10% (*w*/*v*) aluminum chloride solution was added and allowed to react for 5 min. Then, 200 μL of 1 M sodium hydroxide was introduced to complete the color development. The absorbance of the resulting mixture was recorded at 510 nm using a GENESYS™ 180 UV–Visible spectrophotometer (Thermo Fisher Scientific, Waltham, MA, USA). TFC was expressed as milligrams of quercetin equivalents (mg QE) per gram of microcapsule (powder), based on the standard calibration curve using Equation (9).

(9) $$\mathrm{TFC}\;(\mathrm{mg}\;\mathrm{QE}/g\;\mathrm{powder})=\frac{\left(QE\times EV\right)}{\left(ME\right)}$$

where QE is quercetin (mg/mL) as a standard, EV is the extract volume (mL), and ME is mass of the extract microcapsule powder.

#### 2.5.4. Major Encapsulated Compounds Detection

To analyze the major flavonoid compounds, the extracted core materials were subjected to high-performance liquid chromatography (HPLC) using an Agilent 1260 system (Agilent 1260, Agilent Technologies, Santa Clara, CA, USA) with a Zorbax SB-C18 column (250 mm × 4.6 mm, 5 μm). A gradient elution method adapted from Fahrudin et al. [8] was employed, using 0.1% phosphoric acid (solvent A) and acetonitrile (solvent B) as the mobile phase. The elution began with 20% A/80% B, maintained until 35 min, gradually shifted to 80% A/20% B by 45 min, and held until 50 min. The flow rate was 1 mL/min, and detection was performed at 256 nm. A 10 μL sample was injected, and compound identification was based on retention time and UV spectra compared to authentic standards. Quantification of pinostrobin and pinocembrin was based on calibration curves ranging from 0.5 to 50 ppm and expressed as mg/g microcapsule (powder).

#### 2.5.5. Encapsulation Efficiency and Surface Phenolic Analysis

Encapsulation efficiency was evaluated using the methodology of Velazquez-Martinez et al. [19]. A quantity of 200 mg of microcapsules were combined with 1 mL of acetonitrile and 1 mL of a 50:8:42 *v*/*v*/*v* methanol, acetic acid, and water solution to dissolve the microcapsule covering and liberate the contained phenolic chemicals. The solution was gathered for examination of TPC following centrifugation. To extract surface-exposed phenolic chemicals for surface phenolic content (SPC), 200 mg of microcapsules were combined with a 50:50 *v*/*v* ethanol and methanol solution. After centrifuging the samples for 5 min at room temperature at 3500 rpm and vortexing them for 1 min, they were filtered through 0.45 µm syringe filters. The Folin–Ciocalteu technique was then used to determine total phenolic content. Equation (10) was used to determine the encapsulation efficiency, or the percentage of phenolic compounds successfully kept inside the microcapsules.

(10) $$\mathrm{Encapsulation}\;\mathrm{Efficiency}\;(\%)=\frac{\left(TPC-SPC\right)}{TPC}$$

### 2.6. Microcapasule Thermal Stability Analysis

Thermal stability was assessed using a modified protocol based on [20], employing differential scanning calorimetry (DSC) (DSC 8500, PerkinElmer, Shelton, CT, USA). Approximately 3 mg of each spray-dried sample was weighed into an open aluminum crucible. The samples were equilibrated at 30 °C for 1 min and then heated to 200 °C at a rate of 10 °C/min under a nitrogen flow of 20 mL/min. An empty aluminum crucible was used as a reference. The glass transition temperature (T_g_) was determined as the midpoint of the step change in the heat flow baseline, corresponding to the transition from a glassy to a rubbery state. The peak temperature (T_peak_) represents the maximum point of the endothermic event associated with structural transitions such as matrix relaxation or softening. Additionally, the associated enthalpy change (ΔH) was recorded to quantify the thermal energy absorbed during the transition. The melting temperature (T_m_), in the context of DSC is commonly interpreted as either the onset temperature where melting initiates or the peak temperature, where heat absorption is at its maximum during the phase change. These thermal parameters provide insight into the thermal resistance and stability of the microcapsule matrices under processing or storage conditions.

## 3. Results and Discussions

### 3.1. Effects of Wall Materials on Fingerroot Powders Flow Properties

Flowability is a critical factor influencing spray-dried powders’ handling, processing, and application. Flow properties of the microcapsules are shown in Table 3. Formulations incorporating maltodextrin (MD20) exhibited relatively better flowability, indicated by a loose bulk density of 0.37 g/mL, a Hausner ratio of 1.45, and a Carr Index of 31.15%. In contrast, gum arabic (GA20) and combined formulations like MD0.5GA1 demonstrated poorer flowability, with MD0.5GA1 showing a Hausner ratio of 1.64 and Carr Index of 39.01%. MD1GA0.5 showed similar tapped bulk density, Hausner ration, Carr Index, and hence flowability as compared to MD1GA1 (*p* > 0.05). The poor flow characteristics of these formulations are largely attributed to the sticky nature of gum arabic, which leads to increased particle aggregation during drying. This reduces powder cohesiveness and increases interparticle friction, contributing to decreased flowability. Evidence supports that lower hygroscopicity positively correlates with improved flowability, as encapsulated powders that absorb less moisture from the environment tend to maintain better physicochemical characteristics and flow dynamics [21]. Formulations with high cohesiveness are more prone to environmental degradation from moisture, heat, and oxidation, potentially leading to premature release or loss of bioactivity [22]. Stability issues are often related to particle size distribution, surface morphology, and matrix integrity [23]. Formulation adjustments may be necessary to mitigate these challenges, including optimizing wall material ratios or incorporating protective agents [24].

### 3.2. Fingerroot Powder Characteristics of Various Wall Materials

Particle size significantly affects the solubility, dispersibility, and flowability of spray-dried powders. As shown in Table 4, MD0.5GA1 produced the smallest particles (3.11 µm), while MD1GA0.5 resulted in the largest particles (10.94 µm). Smaller particles typically offer higher surface area, improving solubility and reconstitution. The polydispersity index (PDI) is a numerical indicator of the uniformity of particle size distribution in a sample, where lower values reflect more homogeneous systems. In general, PDI values < 0.1 indicate a highly monodisperse system, values between 0.1 and 0.2 are considered monodisperse, and values > 0.2 suggest increasing degrees of polydispersity. In this study, as shown in Table 4, GA20 exhibited the narrowest particle size distribution with a PDI of 0.19, falling within the monodisperse range. This suggests that particles produced with 20% gum arabic as the sole wall material were relatively uniform in size, which may enhance physical stability and performance. Conversely, the highest PDI value was observed in MD0.5GA1 (0.84), indicating a broad size distribution and significant heterogeneity in particle formation. This high degree of polydispersity may be attributed to droplet coalescence or inconsistent solidification dynamics during spray-drying when using a combination of maltodextrin and gum arabic at low concentrations. A PDI approaching 1.0 suggests the system was approaching high polydispersity, which may compromise reproducibility and stability of the microencapsulated product. Therefore, selection of appropriate wall material ratios is critical for achieving uniform particle size distribution, which is essential in applications where functional performance depends on particle uniformity

The volume-weighted mean diameter (D[4,3]), which reflects the average particle size based on volume and emphasizes larger particles, further explains the physical behavior of the powders. GA20 and MD20 had the highest D[4,3] values (4.45 and 4.13 µm, respectively), indicating more large particles that may improve flowability but reduce solubility. In contrast, MD1GA0.5 (1.08 µm) had the lowest D[4,3], suggesting a finer powder with higher solubility potential but possibly lower flowability. These results underscore the importance of balancing particle size characteristics to optimize powder functionality.

Wall material composition and core-to-coating ratios significantly influenced particle size distribution. Formulations with higher gum arabic content, such as MD1GA1, tended to produce larger and more heterogeneous particles, while maltodextrin-dominant formulations yielded smaller, more uniform particles due to their lower viscosity and efficient film-forming properties [25].

Solubility measurement showed that MD1GA0.5 had the highest solubility (99.08%), making it particularly suitable for applications requiring rapid reconstitution [26]. MD0.5GA1 followed closely at 97.80%, remaining effective for most. In terms of hygroscopicity, GA20 exhibited the highest moisture uptake (5.52%) as shown in Table 4, which may promote clumping and reduce shelf life under humid conditions. Conversely, MD20 showed the lowest hygroscopicity (4.17%), indicating better stability. These findings align with trends in caking behavior, where GA20 had the highest caking index (70.12%) and MD1GA0.5 the lowest (39.79%), reinforcing the good flowability and handling performance of the latter.

About moisture content (Table 5), the GA20 formulation exhibited the highest value (11.99% db), followed by MD0.5GA1 (11.26% db), which may be attributed to the hygroscopic nature of gum arabic. Conversely, MD1GA0.5 had the lowest moisture content (8.46% db), suggesting better drying efficiency or a reduced water retention capacity in this formulation. Water activity (a_w_) values were relatively similar across all samples, ranging from 0.54 to 0.56, and remained below the threshold (a_w_ < 0.60) necessary for microbial stability in dry powders (Table 5). Notably, no statistically significant differences in a_w_ were observed among the formulations, indicating consistent drying performance. These findings suggest that the composition of wall materials plays a crucial role in determining the physical characteristics of the resulting microparticles, particularly concerning moisture, retention, and visual appearance. The combination of maltodextrin and gum arabic, especially in equal proportions, appears to balance brightness and moisture content effectively, an advantage for applications requiring stable and visually appealing powder forms [27]. Moisture content and water activity are critical factors influencing the flow properties and storage stability of spray-dried powders. Furthermore, spray-drying parameters such as feed flow rate and inlet temperature significantly influence both moisture content and water activity. Higher inlet temperatures typically lead to greater water evaporation, resulting in powders with lower moisture levels and enhanced stability [28]. In this context, the slightly elevated moisture content observed in some formulations, such as GA20 and MD0.5GA1, may also reflect the combined effects of the wall materials’ intrinsic water-binding properties, especially gum arabic and suboptimal spray-drying parameters that require further optimization to improve drying efficiency.

### 3.3. Bioactive Content in Fingerroot Microcapsules

The bioactive compound contents (pinostrobin and pinocembrin) of the encapsulated powders, shown in Table 6, varied significantly with wall material composition. The highest total phenolic content (TPC) was observed in the MD1GA0.5 formulation (pinostrobin 190.21 mg GAE/g powder), followed by MD0.5GA1 and MD1GA1, indicating that balanced combinations of maltodextrin (MD) and gum arabic (GA) enhance phenolic retention during spray-drying. In contrast, GA20 (gum arabic alone) showed the lowest TPC (138.34 mg GAE/g powder), likely due to GA’s lower protective capacity for hydrophilic phenolics. For total flavonoid content (TFC), MD20 (maltodextrin alone) had the highest value (167.91 mg QE/g powder), suggesting MD’s greater ability to retain flavonoids, possibly due to hydrophobic interactions with their aromatic structures [29]. These results suggest that phenolic compounds benefit from the combined emulsifying and stabilizing properties of GA and MD, while flavonoids are better preserved in MD-dominant matrices [30,31].

Encapsulation efficiency (EE), as shown in Table 6, further supports this relationship. The highest EE was recorded in MD0.5GA1 (78.68%), along with MD1GA0.5 (76.92%), while GA20 had the lowest (67.86%), reinforcing maltodextrin’s role in improving core material retention. These findings align with studies showing that mixed wall systems optimize both protection and entrapment of bioactives [32]. Notably, surface phenolic content (SPC) was inversely related to EE. MD1GA1, with moderate EE, had the highest SPC (46.18 mg GAE/g powder), indicating possible leakage or incomplete encapsulation. MD1GA0.5 exhibited slightly higher SPC and EE (*p* > 0.05). In contrast, MD0.5GA1 had lower SPC (40.17 mg GAE/g powder), suggesting more effective entrapment within the particle matrix. Although surface phenolics can enhance antioxidant activity, excessive amounts may accelerate degradation or release during storage [33].

Processing conditions also play a crucial role in bioactive retention. Elevated inlet temperatures (e.g., ≥175 °C) have been associated with significant degradation of phenolics and flavonoids, whereas moderate temperatures below 150 °C help preserve their stability. Additionally, feed rate adjustments influence EE, where higher feed rates can reduce thermal exposure, thus minimizing degradation [34].

Table 7 shows the concentrations of pinostrobin and pinocembrin in encapsulated extracts using various maltodextrin (MD) and gum arabic (GA) formulations. Among all samples, the highest retention of both compounds was achieved in MD1GA0.5 (pinostrobin 53.65 mg/g powder; pinocembrin 98.45 mg/g powder) and MD0.5GA1 (pinostrobin 50.72 mg/g powder; pinocembrin 97.08 mg/g powder), with statistically similar values (denoted by the letter *a*), indicating optimal encapsulation efficiency when MD and GA are combined in appropriate ratios. In addition, MD1GA1 also exhibited significantly higher compound retention than the single-material formulations, especially for pinocembrin (92.16 ± 0.37 mg/g powder), though slightly lower than MD1GA0.5 and MD0.5GA1. When comparing single-material carriers, GA20 (gum arabic alone) resulted in higher pinostrobin content (41.47 ± 0.08 mg/g powder) than MD20 (33.21 mg/g powder), showing gum arabic’s better compatibility with polar phenolic compounds. However, contrary to initial assumptions, GA20 also retained more pinocembrin (87.29 ± 0.18 mg/g powder) than MD20 (81.34 ± 0.05 mg/g powder), suggesting that gum arabic may provide better protection for both compounds under the tested conditions.

These trends suggest that the synergistic use of MD and GA enhances retention by combining GA’s emulsifying properties and MD’s film-forming capabilities, forming a more effective barrier during spray-drying. The highest values in dual-material formulations also align with findings that such combinations better protect compounds with varying polarities. Furthermore, based on previously reported non-encapsulated extract values [8], these results support the protective role of encapsulation. The relatively higher stability of pinostrobin, which possesses a methoxy group at C-5, may explain its lesser degradation compared to pinocembrin under thermal stress. This dual-material approach not only maximizes encapsulation efficiency but also promotes higher retention of both phenolics and flavonoids [35].

### 3.4. Thermal Stability of Fingerroot Extract Microcapsules

The thermal stability of the encapsulated powders was influenced by the composition of the wall materials, with notable differences observed among the formulations (Table 8). The MD0.5GA1 sample exhibited the highest T_g_-half Cp (95.29 °C) and enthalpy change (∆H: 29.12 J/g), suggesting a better thermal resistance attributed to the synergistic interaction between maltodextrin (MD) and gum arabic (GA), which enhances matrix strength and energy absorption during thermal transitions [36]. The MD1GA0.5 showed similar onset temperature, T_peak_ and T_end_ (*p* > 0.05); nevertheless, its T_g_ was significantly lower (74.82 °C) as compared to MD0.5GA1, suggesting good thermal stability but more sensitive to glass transition. As shown in Table 8, GA20 displayed the lowest thermal parameters (T_g_-half Cp: 81.57 °C; ∆H: 14.52 J/g), indicating reduced stability when GA is used alone. Although onset, peak, and endpoint temperatures remained consistent (~127–130 °C) across samples, the higher ∆H values in MD-GA blends suggest a stronger, more cohesive matrix better suited for applications requiring heat resistance, such as food processing or thermal storage.

The improved thermal performance of MD-GA blends can be attributed to their complementary properties. MD offers structural rigidity and moisture resistance, while GA contributes emulsifying capacity and matrix flexibility [37,38]. These interactions foster stable encapsulation systems capable of withstanding thermal stress and reducing degradation of bioactive compounds [39]. In contrast, the lower ∆H of GA-only powders may lead to reduced thermal resilience, as less energy is needed for their phase transition [40]. Given the link between thermal stability and long-term bioactive retention, optimizing MD/GA ratios is essential not only for structural integrity but also for enhancing the functional longevity of encapsulated compounds [41]. It should be noted that the DSC results primarily reflect the thermal properties of the encapsulating matrix rather than the thermal stability of the bioactive compounds themselves. Nevertheless, this data is valuable for determining the applicability of microcapsules in thermally processed food systems. Future studies will include targeted thermal degradation analyses of encapsulated pinostrobin and pinocembrin to more accurately assess bioactive protection.

### 3.5. Morphology of Fingerroot Extract Microcapsules

**Figure 1:**
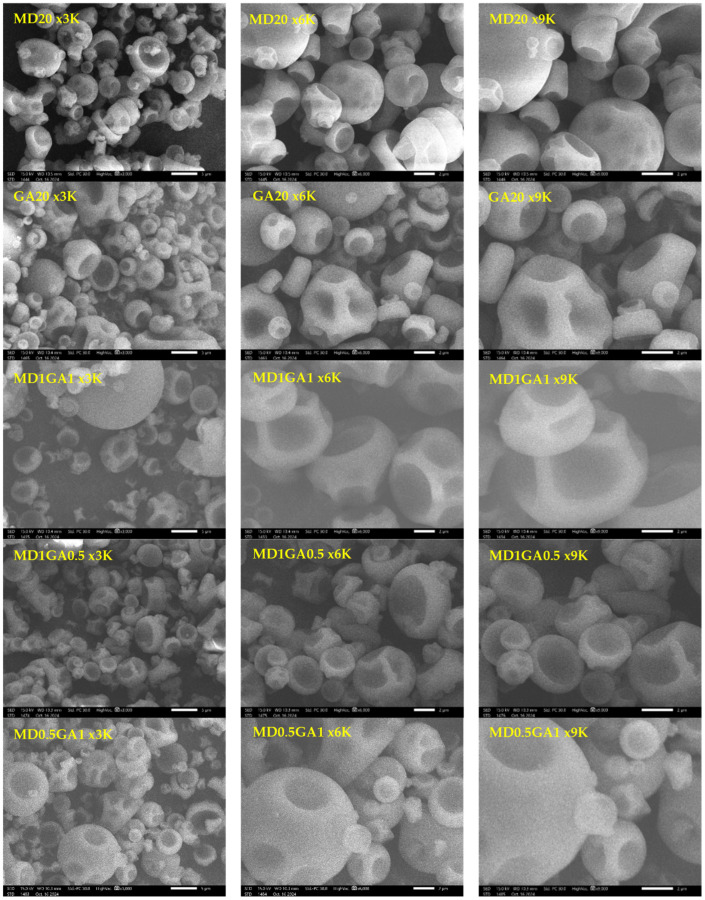
Morphology of fingerroot extract microcapsules.

The morphology of spray-dried encapsulated fingerroot powder by the types and ratios of wall materials, specifically maltodextrin and gum arabic, is shown in Figure 1. The spherical shapes observed in many spray-dried powders are primarily attributed to the rapid evaporation of water from droplets during the drying process, which creates consistent, solid particles [42]. As shown for MD20, microcapsules are more spherical than other treatments due to the rapid release of water from the MD bonding by evaporation. This process can vary based on the material’s surface activity and the drying conditions, leading to phenomena such as bowl-shaped capsules, which can be seen in MD1GA0.5 and other microcapsules containing GA in the wall formulations. This may arise from uneven drying rates or variations in wall material viscosity [43,44]. Furthermore, GA film can have high stretching ability during water evaporation from inside the particle, causing microcapsules to expand. Once the water evaporates, the film can shrink in its rubbery state before turning into a dry, glassy state. As a result, microcapsules appear irregular and less spherical. Encapsulation efficiency and stability are further enhanced when an appropriate wall material composition is used, as it influences not only the morphology but also provides a protective shell that minimizes degradation of the core compounds [45]. 

## 4. Conclusions

This study demonstrated the successful microencapsulation of *Boesenbergia rotunda* extract using maltodextrin and gum arabic as wall materials via spray-drying. The physicochemical characteristics of the resulting powders were influenced significantly by the MD: GA ratio. Among all formulations, the use of MD:GA at 1:1 ratio offered a balanced profile of favorable physicochemical properties, high solubility, moderate hygroscopicity, good flowability, and acceptable encapsulation efficiency, without any single parameter being the highest. This ratio may be considered a suitable compromise for functional powder development. SEM analysis showed uniform spherical morphology, and HPLC confirmed the retention of key bioactive compounds. These results underscore the potential of this formulation for developing stable, functional powder ingredients enriched with fingerroot bioactives for use in food, beverage, and pharmaceutical applications. Future research will investigate the in vitro stability, release profile, and delivery behavior of the encapsulated bioactive compounds in relevant food systems, with a particular focus on compound-specific solubility and release under simulated gastrointestinal conditions. Although this study assessed overall powder solubility, it did not directly examine the behavior of individual bioactives, an essential step toward predicting delivery efficiency and in vivo bioavailability.

## Figures and Tables

**Table 1:** Composition of feed mixture carrier agents used for spray-dried microencapsulation.

Sample	Maltodextrin (% *w*/*v*)	Gum Arabic (% *w*/*v*)	Wall Material Ratio (MD:GA)
MD20	20	0	1:0
GA20	0	20	0:1
MD1GA1	10	10	1:1
MD1GA0.5	13.33	6.66	2:1
MD0.5GA1	6.66	13.33	1:2

**Table 2:** Relationship between Carr index and Hausner ratio with powder flow property [11].

Carr Index	Flow Property	Hausner Ratio
≤10	Excellent	1.00–1.11
11–15	Good	1.12–1.18
16–20	Fair	1.19–1.125
21–25	Passable	1.26–1.34
26–31	Poor	1.35–1.45
32–37	Very poor	1.46–1.59
>38	Very, very poor	>1.60

**Table 3:** Effect of wall materials on flow characteristics of encapsulated powder.

Samples	Loose Bulk Density (g/mL)	Tapped Bulk Density (g/mL)	Hausner Ratio	Carr Index	Flowability
MD20	0.37 ± 0.00 ^a^	0.54 ± 0.03 ^bc^	1.45 ± 0.06 ^c^	31.15 ± 2.83 ^c^	Poor
GA20	0.34 ± 0.00 ^b^	0.52 ± 0.01 ^bc^	1.55 ± 0.04 ^b^	35.39 ± 1.82 ^b^	Very poor
MD1GA1	0.34 ± 0.01 ^b^	0.51 ± 0.01 ^c^	1.52 ± 0.01 ^bc^	34.28 ± 0.58 ^bc^	Very poor
MD1GA0.5	0.36 ± 0.00 ^a^	0.53 ± 0.01 ^bc^	1.49 ± 0.06 ^bc^	33.01 ± 2.63 ^bc^	Very poor
MD0.5GA1	0.35 ± 0.00 ^b^	0.58 ± 0.01 ^a^	1.64 ± 0.04 ^a^	39.01 ± 1.49 ^a^	Very, very poor

Different lowercase letters indicate significant differences in each column at *p* ≤ 0.05.

**Table 4:** Mean particle size, particle size distribution (PDI), D[4,3], solubility, hygroscopic behavior, and caking characteristics of encapsulated powder.

Samples	Mean Particle Size (µm)	PDI	D[4,3] (μm)	Hygroscopicity (%)	Powder Caking (%)	Solubility (%)
MD20	6.76 ± 0.39 ^b^	0.43 ± 0.10 ^c^	4.13 ± 0.18 ^a^	4.17 ± 0.13 ^c^	49.35 ± 0.96 ^c^	98.62 ± 0.20 ^b^
GA20	5.24 ± 0.12 ^c^	0.19 ± 0.11 ^d^	4.45 ± 0.12 ^a^	5.52 ± 0.21 ^a^	70.12 ± 2.28 ^a^	98.33 ± 1.44 ^b^
MD1GA1	6.50 ± 0.00 ^b^	0.42 ± 0.04 ^c^	3.15 ± 2.33 ^b^	5.08 ± 0.06 ^b^	59.99 ± 0.66 ^b^	98.70 ± 0.65 ^b^
MD1GA0.5	10.94 ± 2.10 ^a^	0.53 ± 0.39 ^b^	1.08 ± 0.58 ^c^	4.92 ± 0.05 ^b^	39.79 ± 0.51 ^d^	99.08 ± 0.32 ^a^
MD0.5GA1	3.11 ± 0.13 ^d^	0.84 ± 0.22 ^a^	1.42 ± 0.04 ^c^	4.99 ± 0.06 ^b^	56.10 ± 0.49 ^b^	97.80 ± 0.37 ^b^

Different lowercase letters indicate significant differences in each column at *p* ≤ 0.05.

**Table 5:** Moisture content and water activity of encapsulated powder.

Samples	Moisture Content (% db)	Water Activity
MD20	10.57 ± 0.40 ^b^	0.55 ± 0.02 ^ab^
GA20	11.99 ± 0.84 ^a^	0.56 ± 0.00 ^a^
MD1GA1	8.69 ± 0.94 ^c^	0.55 ± 0.01 ^ab^
MD1GA0.5	8.46 ± 0.66 ^c^	0.54 ± 0.01 ^ab^
MD0.5GA1	11.26 ± 0.54 ^ab^	0.54 ± 0.00 ^b^

Different lowercase letters indicate significant differences in each column at *p* ≤ 0.05.

**Table 6:** Wall material effects on encapsulation efficiency, total phenolic, surface phenolic, and flavonoid content of encapsulated powders.

Samples	EE (%)	TPC (mg GAE/g Powder)	SPC (mg GAE/g Powder)	TFC (mg QE/g Powder)
MD20	74.23 ± 2.06 ^c^	163.02 ± 15.51 ^b^	41.80 ± 2.35 ^bc^	167.91 ± 14.35 ^a^
GA20	67.86 ± 2.08 ^d^	138.34 ± 4.83 ^c^	44.49 ± 3.72 ^ab^	143.07 ± 3.31 ^b^
MD1GA1	75.06 ± 1.11 ^bc^	185.51 ± 8.79 ^a^	46.18 ± 0.88 ^a^	157.44 ± 1.87 ^ab^
MD1GA0.5	76.92 ± 1.03 ^ab^	190.21 ± 6.59 ^a^	43.87 ± 1.30 ^ab^	148.83 ± 12.82 ^b^
MD0.5GA1	78.68 ± 0.65 ^a^	188.52 ± 5.12 ^a^	40.17 ± 1.03 ^c^	146.84 ± 3.09 ^b^

Different lowercase letters indicate significant differences in each column at *p* ≤ 0.05.

**Table 7:** Major compounds of the encapsulated powder.

Samples	Pinostrobin (mg/g Powder)	Pinocembrin (mg/g Powder)
MD20	33.21 ± 0.12 ^c^	81.34 ± 0.05 ^b^
GA20	41.47 ± 0.08 ^b^	87.29 ± 0.18 ^b^
MD1GA1	45.89 ± 0.12 ^b^	92.16 ± 0.37 ^a^
MD1GA0.5	53.65 ± 1.03 ^a^	98.45 ± 0.20 ^a^
MD0.5GA1	50.72 ± 0.65 ^a^	97.08 ± 0.14 ^a^

Different lowercase letters indicate significant differences in each column at *p* ≤ 0.05.

**Table 8:** Thermal parameters of fingerroot extract microcapsules determined by DSC.

Samples	T_g_-half Cp (°C)	Onset (°C)	T_peak_ (°C)	ΔH (J/g)	T_End_ (°C)
MD20	91.9 ± 0.21 ^b^	127.12 ± 1.90 ^a^	127.92 ± 1.42 ^a^	25.48 ± 6.43 ^ab^	128.69 ± 1.57 ^a^
GA20	81.57 ± 0.87 ^d^	127.51 ± 3.97 ^a^	128.53 ± 3.80 ^a^	14.52 ± 7.57 ^c^	128.94 ± 3.71 ^a^
MD1GA1	85.98 ± 2.53 ^c^	127.51 ± 1.34 ^a^	128.56 ± 1.34 ^a^	27.35 ± 1.37 ^a^	129.39 ± 1.40 ^a^
MD1GA0.5	74.82 ± 1.58 ^e^	128.52 ± 1.34 ^a^	129.36 ± 1.03 ^a^	16.72 ± 4.13 ^bc^	129.71 ± 0.95 ^a^
MD0.5GA1	95.29 ± 2.74 ^a^	129.11 ± 5.00 ^a^	130.53 ± 4.57 ^a^	29.12 ± 1.50 ^a^	129.80 ± 2.56 ^a^

T_g_-half Cp: Glass transition midpoint. Onset: Initial temperature of thermal event. T_peak_: Peak temperature. ΔH: Enthalpy change. T_End_: End temperature of thermal event. Different lowercase letters indicate significant differences in each column at *p* ≤ 0.05.

## Data Availability

The original contributions presented in this study are included in the article. Further inquiries can be directed to the corresponding author.
